# A target based approach identifies genomic predictors of breast cancer patient response to chemotherapy

**DOI:** 10.1186/1755-8794-5-16

**Published:** 2012-05-11

**Authors:** Robin M Hallett, Gregory Pond, John A Hassell

**Affiliations:** 1 Department of Biochemistry and Biomedical Sciences, Centre for Functional Genomics, McMaster University, 1200 Main Street West, Hamilton, Ontario, L8N 3Z5, Canada; 2 Department of Oncology, Juravinski Hospital and Cancer Centre, 711 Concession Street, Hamilton, Ontario, L8V 1C3, Canada; 3 Centre for Functional Genomics, McMaster University, 1280 Main St West, Hamilton, Ontario, L8S 4K1, Canada

**Keywords:** Gene expression profiling, Breast cancer, Chemotherapy response, Gene signatures, Chemotherapy

## Abstract

**Background:**

The efficacy of chemotherapy regimens in breast cancer patients is variable and unpredictable. Whether individual patients either achieve long-term remission or suffer recurrence after therapy may be dictated by intrinsic properties of their breast tumors including genetic lesions and consequent aberrant transcriptional programs. Global gene expression profiling provides a powerful tool to identify such tumor-intrinsic transcriptional programs, whose analyses provide insight into the underlying biology of individual patient tumors. For example, multi-gene expression signatures have been identified that can predict the likelihood of disease reccurrence, and thus guide patient prognosis. Whereas such prognostic signatures are being introduced in the clinical setting, similar signatures that predict sensitivity or resistance to chemotherapy are not currently clinically available.

**Methods:**

We used gene expression profiling to identify genes that were co-expressed with genes whose transcripts encode the protein targets of commonly used chemotherapeutic agents.

**Results:**

Here, we present target based expression indices that predict breast tumor response to anthracycline and taxane based chemotherapy. Indeed, these signatures were independently predictive of chemotherapy response after adjusting for standard clinic-pathological variables such as age, grade, and estrogen receptor status in a cohort of 488 breast cancer patients treated with adriamycin and taxotere/taxol.

**Conclusions:**

Importantly, our findings suggest the practicality of developing target based indices that predict response to therapeutics, as well as highlight the possibility of using gene signatures to guide the use of chemotherapy during treatment of breast cancer patients.

## Background

Oncologists are faced with the challenging task of selecting the most effective therapies for individual cancer patients to achieve the best possible outcome. Indeed, the latter is the central goal of personalized cancer medicine. Trastuzumab is one of the best examples illustrating the importance of tailoring treatment to the characteristics of an individual’s tumor. In the absence of patient selection only 10% of breast cancer patients derive clinical benefit from trastuzumab treatment [1]. However, when patients are selected for trastuzumab therapy based on ERBB2/HER2 gene amplification, their response rate rises to as high as 50%. The development of global gene expression profiling technologies, such as DNA microarrays, has provided additional avenues to identify the molecular features of tumors that are associated with clinical variables, such as tumor grade or outcome. In fact, sets of genes, commonly called gene signatures, have been identified that predict patient prognosis, and are already used in various clinical settings [2-8].

Many current studies focus on identifying similar gene signatures to guide the selection of appropriate chemotherapy regimens [9-12]. However, gene signatures that predict tumor sensitivity or resistance to chemotherapy are not currently clinically available. The development and clinical implementation of gene signatures that predict response to commonly used chemotherapeutic agents could facilitate selecting the most efficacious therapeutic regimen given the molecular characteristics of an individual’s tumor. Furthermore, therapy-predictive gene signatures could ensure patients do not receive ineffective and potentially deleterious chemotherapeutic regimens.

Measuring the inherent chemosensitivity of a tumor can be accomplished by assessment of pathological response following neoadjuvant treatment with a given treatment regimen. In this fashion, patients in which no invasive or metastatic breast cancer can be detected following treatment are classified as having achieved complete pathological response (pCR), whereas patients that fail to achieve pCR are classified as having residual disease (RD). Importantly, neoadjuvant chemotherapy has been found to be as efficacious as chemotherapy given in the adjuvant setting, and patients who achieve complete pathological response after neoadjuvant intervention generally have an excellent probability of experiencing long-term survival [13-15]. Taken together, these data suggest that response to neoadjuvant chemotherapy (pCR/RD) provides a relevant clinical model to develop and validate gene signature based predictors of breast tumor response to chemotherapy.

We sought to test whether TOP2A and β-tubulin transcript expression indices could predict response to commonly used chemotherapeutic agents, as the protein products of these genes represent the respective molecular targets of commonly used anthracycline- and taxane-related drugs [16-18]. We hypothesized that such target based expression indices would provide a biologically comprehensive measurement of either TOP2A or β-tubulin activity in a patient’s tumor, and thus its likely dependence on either of these targets. Importantly, these analyses establish an effective method for identifying predictive drug response signatures, and highlight the use of predictive gene signatures to guide the selection of anthracycline and taxane based chemotherapy regimens for breast cancer patients.

## Results

### TOP2A and β-tubulin expression are associated with complete pathological response in breast cancer patients treated with chemotherapy

**Figure 1:**
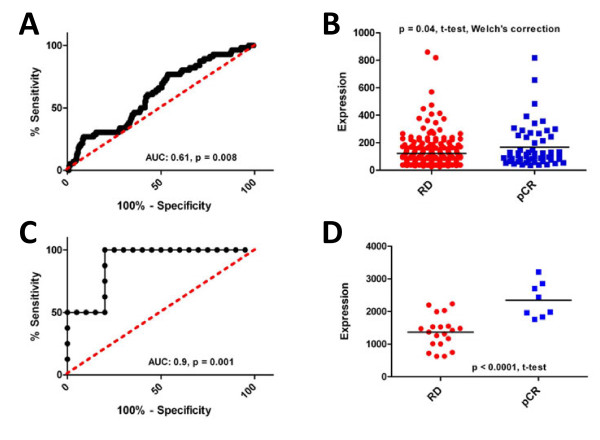
**TOP2A and β-tubulin transcript levels predict response to anthracycline or taxane containing chemotherapy, respectively.A**) ROC analysis of TOP2A transcript levels and response to TFAC neoadjuvant chemotherapy (AUC: 0.61, *p* = 0.008). **B**) Expression levels of TOP2A transcripts in patients who experienced pCR or had RD after neoadjuvant chemotherapy ( *p* = 0.04, t-test). **C**) ROC analysis of β-tubulin transcript levels and response to neoadjuvant docetaxel chemotherapy (AUC: 0.9, *p* = 0.001). **D**) Expression levels of β-tubulin transcripts in patients who experienced pCR or had RD after neoadjuvant docetaxel chemotherapy ( *p* < 0.0001, t-test).

As proof-of-principle that target expression could be linked to chemotherapy response, we tested whether transcripts of TOP2A were associated with pCR in a relatively large (GSE21094, n = 278, n = 56 pCR, n = 222 RD) cohort of breast cancer patients treated with a neoadjuvant chemotherapy regimen that contained an anthracycline (TFAC; paclitaxel [T], 5-FU [F], adriamycin [A] and cyclophosphamide[C]). TOP2A transcript levels were associated with increased response to chemotherapy (Figure 1A, AUC: 0.61, *p* = 0.008), and the mean expression of TOP2A transcripts was higher in patients who showed a complete pathological response than those who displayed residual disease after treatment (Figure 1B, RD: 122.5, pCR: 167.7, *p* = 0.04, t-test, Welch’s correction). Importantly, these data suggested that elevated expression levels of TOP2A transcripts are associated with response to anthracycline based chemotherapy regimens, and are consistent with previous studies that link TOP2A expression with response to anthracycline therapy [19-21]. To extend these findings, we also tested whether β-tubulin expression was associated with pCR in a cohort of breast cancer patients treated with docetaxel. β-tubulin transcript levels were linked with increased response to docetaxel (Figure 1C, AUC: 0.9, *p* = 0.001), and the mean expression of β-tubulin transcripts was higher in complete responders than those whose tumors did not achieve a complete response (Figure 1D, RD: 2344, pCR: 1364, *p* < 0.0001, t-test). Indeed, these findings are consistent with previous studies that link β-tubulin expression with sensitivity to taxane chemotherapy [22]. In total, these experiments suggest that target expression is associated with response to their chemotherapeutic agent counterparts.

### The TOP2A index is associated with complete pathological response in breast cancer patients treated with anthracyclines

**Figure 2:**
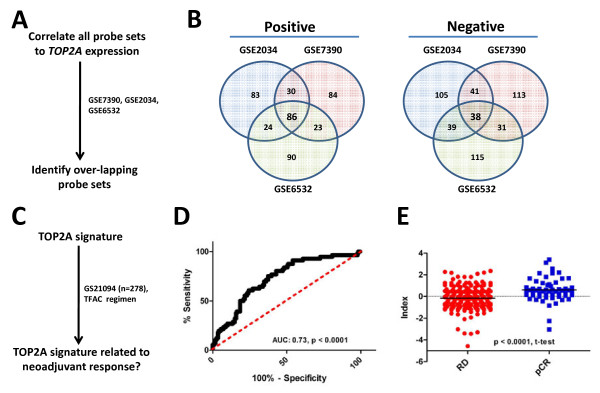
**TOP2A index predicts response to anthracycline containing chemotherapy. A)** Experimental strategy to identify the TOP2A index. **B**) TOP2A co-expressed probesets within the three discovery cohorts. **C**) Experimental strategy to validate the TOP2A index. **D**) ROC Analysis of TOP2A index scores and response to T/FAC neoadjuvant chemotherapy (AUC: 0.73, *p* < 0.0001). E) TOP2A index scores of patients who experienced pCR or had RD after neoadjuvant chemotherapy ( *p* < 0.0001, t-test).

**Figure 3:**
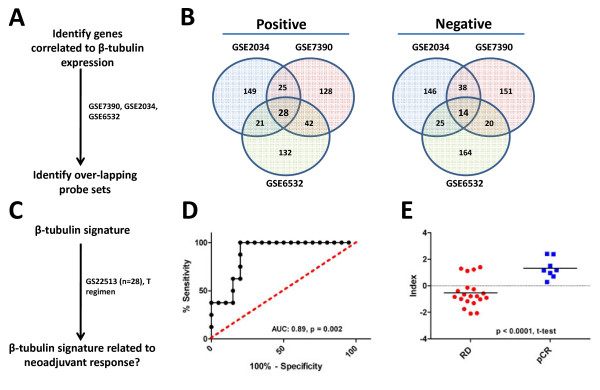
**β-tubulin index predicts response to docetaxel chemotherapy.A**) Experimental strategy to identify the β-tubulin index. **B**) β-tubulin co-expressed probesets within the three discovery cohorts. **C**) Experimental strategy to validate the β-tubulin index. **D**) ROC analysis of β-tubulin index scores and response to docetaxel neoadjuvant chemotherapy (AUC: 0.89, *p* = 0.002). E) β-tubulin index scores in patients that experienced pCR or had RD after neoadjuvant chemotherapy ( *p* < 0.0001, t-test).

Our previous observations suggested the expression of transcripts encoding the protein targets of therapeutic agents was associated with response to these same therapies. To extend these findings, we calculated a TOP2A transcript expression index by identifying genes whose expression displayed either positive or negative correlation to the expression of TOP2A transcripts in 3 independent publicly available data sets (GSE2034, GSE7390, GSE6532) (Figure 2A). In short, we measured the correlation of all probe sets to TOP2A expression and selected those that ranked among the top and bottom 1% of all probe sets in each data set. The latter approach identified 124 TOP2A associated probe sets, 86 that displayed positive association to TOP2A transcripts and 34 that were negatively associated with TOP2A transcripts (Figure 2B Additional file 1: Table S1). Using the same 278 patient cohort we employed previously, the TOP2A index was significantly associated with pCR (Figure 2D, AUC: 0.73, *p* < 0.0001) and the TOP2A index scores were higher in patients who demonstrated a pCR than those who retained RD after treatment (Figure 2E, RD:–0.15, pCR: 0.60, *p* < 0.0001, t-test,). Notably, the TOP2A index included genes linked to DNA repair, including EXO1[23], ERCC6L[24,25], and RAD51[26], suggesting that the TOP2A index has a functional connection to the mechanisms of action of anthracycline drugs. To look for similarities between the TOP2A index and other predictors of chemotherapy response, we examined whether TOP2A index probes were also present within the DLDA30 predictor. Importantly, the DLDA30 is a validated gene expression based predictor of response to taxanes and anthracyclines, as well as to 5-fluorouracil and cyclophosphamide [9,10,12]. Interestingly, only one probe set from the TOP2A index also comprised an element in the DLDA30 predictor, suggesting minimal overlap between these two gene expression based predictors. Taken together, these data provide proof-of- principle that a target based expression index can predict response to small molecules that inhibit the activity of the given target under investigation. Importantly, we show here that the TOP2A index was associated with complete pathological response in breast cancer patients treated with an anthracycline containing chemotherapy regimen in the neoadjuvant setting. A β-tubulin index is associated with complete pathological response in breast cancer patients treated with taxanes Given the development of the TOP2A index, we explored the possibility of identifying and developing a similar index to predict response to taxane drugs (Figure 3A). In a similar fashion as reported above, we identified an expression index of β-tubulin associated transcripts. β-tubulin is the molecular target of taxane drugs, a commonly used class of chemotherapeutic drugs that includes docetaxel and paclitaxel (Taxotere® and Taxol® respectively)[27-29]. In short, we identified 42 β-tubulin transcript-associated probe sets, 28 that displayed positive association with β-tubulin transcripts and 14 that were negatively associated with β-tubulin transcript levels (Figure 3B). We evaluated the association between the β-tubulin index and response to docetaxel using a 14 patient (profiled in replicate, n = 28, RD: 20, pCR: 8) cohort that was treated with neoadjuvant docetaxel (Figure 3C). The β-tubulin index was associated with complete pathological response (Figure 3D, AUC: 0.89, *p* = 0.002) and β-tubulin index scores were higher in patients who achieved complete pathological response than those who experienced residual disease after docetaxel therapy (Figure 3E, RD: -0.5, pCR: 1.3, *p* < 0.0001, t-test). Interestingly, the β-tubulin index included genes linked to cytoskeleton processes, including TPX2[30], and DBN1[31], suggesting a linkage between the β-tubulin index and the mechanism of action of taxanes. As completed previously, we also looked for similarities between the β-tubulin index and the DLDA30 predictor. Notably, no probe sets from the β-tubulin index comprised an element within the DLDA30 predictor, suggesting minimal overlap between these two gene expression based predictors. In total, these data provide evidence that target based expression indices can predict response to neoadjuvant chemotherapy. In each case, the area under curve (AUC) was significantly greater than 0.5, confirming the predictive capacity of the indices, as well as the overall validity of the approach.

### Combining indices is predictive of response to multi-agent chemotherapy

**Figure 4:**
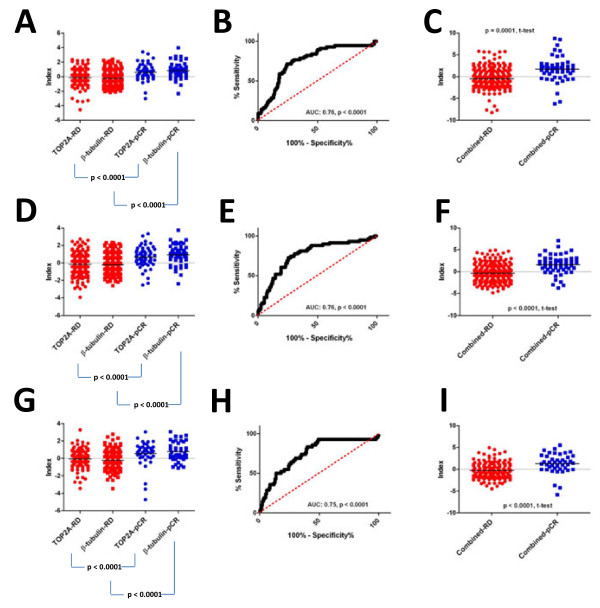
**TOP2A and β-tubulin combination index predicts response to multi-agent anthracycline/taxane chemotherapy.A**) Individual target index scores ( **A**: TOP2A index, T: β-tubulin index) in patients who experienced complete pathological response or had residual disease after TFAC neoadjuvant chemotherapy (GSE21094, *p* < 0.0001 both cases, t-test). B) ROC analysis of combination index scores and response to TFAC neoadjuvant chemotherapy (AUC: 0.78, *p* < 0.0001). **C**) Combination index scores in patients who experienced pCR or had RD after neoadjuvant chemotherapy ( *p* < 0.0001, t-test). **D**) Individual target index scores of patients who experienced pCR or had RD after AT neoadjuvant chemotherapy (GSE25055, *p* < 0.0001 both cases, t-test). **E**) ROC Analysis of combination index scores and response to AT neoadjuvant chemotherapy for GSE25055 (AUC: 0.78, *p* < 0.0001). **F**) Combination index scores of patients who experienced pCR or had RD after AT neoadjuvant chemotherapy ( *p* <0.0001, t-test). **G**) Individual target index scores of patients who experienced pCR or had RD after AT neoadjuvant chemotherapy (GSE25065, *p* < 0.0001 both cases, t-test). **H**) ROC analysis of combination index scores and response to AT neoadjuvant chemotherapy for GSE25065 (AUC: 0.75, *p* < 0.0001). **I**) Combination index scores of patients who experienced complete pathological response or had residual disease after AT neoadjuvant chemotherapy ( *p* < 0.0001, t-test).

Neoadjuvant chemotherapy generally comprises multiple different chemotherapeutic agents. To determine whether we could combine our individual target indices and predict response to multi- agent chemotherapy we tested the TOP2A and β-tubulin indices as a combination index with the 278–patient cohort (TFAC), as well as with two different cohorts of patients treated with neoadjuvant therapy comprising an anthracycline and a taxane (AT) (GSE25055 [n = 310] & GSE25065 [n = 198]). The individual TOP2A and β-tubulin indices were associated with response among the TFAC-treated patients, and patients who experienced pCR had significantly higher individual index scores than those who retained residual disease (Figure 4A, *p* < 0.0001 both cases, t-test). After combining the indices for the 278 TFAC-treated patient cohort, we observed a significant association between the combined indices and tumor response (Figure 4B, AUC: 0.76, *p* < 0.0001), and the combined index scores were significantly higher in responders (pCR) than non-responders (RD) (Figure 4C, RD:–0.4, pCR: 1.7, *p* < 0.0001, t-test). Interestingly, the AUC value for the combined TOP2A and β-tublin index was nominally higher (AUC: 0.78) than that produced by the TOP2A index alone (Figure 2D, AUC:0.73). We also tested the TOP2A and β-tubulin combination index on an additional two independent cohorts of patients treated with AT neoadjuvant chemotherapy (GSE25055 [n = 310] & GSE25065 [n = 198]). In the 310-patient cohort the individual TOP2A and β-tubulin index scores were significantly higher in responders than non- responders (Figure 4D, *p* < 0.0001 both cases, t-test), the combination of the TOP2A and β-tubulin indices were associated with increased response to treatment (Figure 4E, AUC: 0.76, *p* < 0.0001), and the TOP2A/β-tubulin combination index scores were higher in responders than non-responders (Figure 4F, RD: −0.4, pCR: 1.6, *p* < 0.0001, t-test). We made similar observations in the 198-patient cohort, where individual TOP2A and β-tubulin index scores were significantly higher in responders than in nonresponders (Figure 4G, *p* < 0.0001 both cases, t-test), and the combination index score was associated with complete pathological response (Figure 4H, AUC: 0.75, *p* < 0.0001). Again, combined index scores were higher in responders than in non-responders (Figure 4I, RD:–0.3, pCR: 1.3, *p* < 0.0001, t-test). Taken together these data reveal the feasibility of combining individual target indices to predict response to multi-agent chemotherapy regimens.

### TOP2A and β-tubulin indices are more accurate than a similarly derived proliferation index

**Figure 5:**
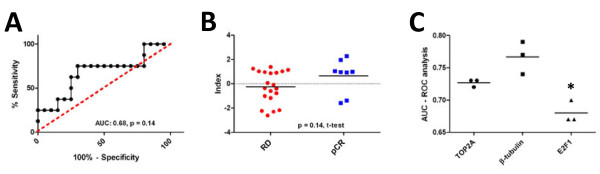
**TOP2A and β-tubulin indices are more accurate than a similarly derived proliferation index.A**) ROC analusis of TOP2A index and patient response to neoadjuvant docetaxel therapy ( *p* = 0.14). **B**) TOP2A index scores of patients who experienced pCR or had RD after neoadjuvant docetaxel chemotherapy ( *p* =0.14, t-test). C) The TOP2A and β-tubulin indices outperform an E2F1-derived proliferation at predicting response to neoadjuvant chemotherapy in multiple patient datasets (* *p* <0.05, ANOVA, Tukey’s test).

Previous work suggests that the prognostic/predictive power of many breast cancer gene expression signatures is derived from their capacity to measure proliferation [2,4,7,32-36]. Hence, it is possible that the TOP2A and β-tubulin indices described here predict response based on their capacity to measure proliferation rather than providing a biologically relevant measurement of target. This is particularly relevant to the TOP2A index, as the protein product of the TOP2A gene is directly involved in DNA synthesis. To address this issue we first tested the capacity of the TOP2A index to predict response in the docetaxel-only treated cohort. In this fashion, the capacity of the TOP2A index to predict response to docetaxel would suggest that the predictive capacity of the TOP2A index is not target specific and may measure a more general phenomenom related to chemotherapy sensitivity, such as proliferation. Within this patient cohort, the TOP2A index was not significantly associated with patient response to docetaxel therapy (Figure 5A&B, AUC: 0.68, *p* = 0.14, RD:–0.26, pCR: 0.65, *p* = 0.12, t-test). Indeed, these results suggest that the predictive capacity of the TOP2A index is based on measurement of target rather than proliferation. We also generated a ‘proliferation index’ built using the same methodology as the TOP2A and β-tubulin indices, around the well characterized proliferation gene E2F1 [8,37-41]. As expected, the E2F1 index was related to chemotherapy response in the 3 large cohorts of breast cancer patients 3 tested previously (GSE21094, GSE25055, GSE25065) ( Additional file 2: Figure S1). Importantly, these results indicate the gene expression signatures that measure proliferation are associated with patient response to chemotherapy. However, the performance of the E2F1 predictor was inferior to either the TOP2A and β-tubulin predictors (Figure 5C, *p < 0.05, ANOVA, Tukeys). Taken together, these data suggest that the predictive capacity of the TOP2A index is specific to anthracycline drugs, and moreover, that the TOP2A and β-tubulin indices are superior to gene signatures that measure proliferation.

### Comparison of the TOP2A and β-tubulin indices with clinico-pathologic parameters

Reported genomic tests for response to chemotherapy have generally failed to outperform the predictive capacity of standard clinic-pathological measurements [10]. In this regard we tested whether the TOP2A/β-tubulin combination index was related to tumor response after adjusting for standard clinical pathological variables. Combination of the two previously used data sets (GSE25055 & GSE25065, 20 patients discarded as pCR/RD data was unavailable) yielded a data set comprising 488 patients for the analysis. In a univariate analysis that included estrogen receptor status, tumor grade, nodal status, patient age, as well as the TOP2A/β-tubulin combination index score, only estrogen receptor status (AUC: 0.68, *p* < 0.001), tumor grade (AUC: 0.69, *p* < 0.001) and the combination index score (Range [−5.829, 7.120], AUC: 0.76, *p* < 0.001) were found to be statistically significantly related to tumor response, whereas age and nodal status were not (Table 1). In a multivariate model (AUC: 0.78), the TOP2A/β-tubulin combination index score remained statistically significantly related to tumor response (*p* < 0.001) as well as estrogen receptor status ( *p* = 0.014) and grade ( *p* = 0.016) (Table 1). In the multivariate model the odds ratio for the TOP2A/β-tubulin index was 1.33, which indicated that for each unit increase (i.e. from −3 to −2, from 0 to 1, or from 4 to 5, etc) in the AT index, there was a 1.33 times increase in the odds of a patient experiencing complete pathological response. Importantly, these data suggest that the TOP2A/β-tubulin combination index score was related to patient response to chemotherapy even after adjusting for standard clinical-pathological variables.

**Table 1:** Logistic regression analysis of the GSE25055 & GSE25065 validation set

**Characteristic**	**Odds ratio (95% CI)**	**AUC**	**p-value**
Univariate Analyses			
AT Index	1.57 (1.39, 1.77)	0.76	<0.001
Age (/10 years)	0.84 (0.68, 1.04)	0.55	0.10
ER Status Positive	0.23 (0.14, 0.37)	0.68	<0.001
Grade	5.28 (2.95, 9.46)	0.69	<0.001
Node Positive	1.16 (0.72, 1.88)	0.52	0.54
Multivariate Analyses			
Age (/10 years)	0.89 (0.70, 1.13)	0.78	0.33
ER Status Positive	0.48 (0.27, 0.86)		0.014
Grade	2.24 (1.16, 4.33)		0.016
Node	0.95 (0.54, 1.67)		0.86
AT Index	1.33 (1.14, 1.55)		<0.001

## Discussion

Here we describe the identification of TOP2A and β-tubulin transcript expression indices that predict complete pathological response to neoadjuvant chemotherapy regimens containing anthracycline and taxane drugs. Complete pathological response represents an appropriate clinical endpoint for these studies as patients who experience pCR also experience improved survival compared to those patients who retain RD [13-15]. Notably, TOP2A or β-tubulin, the respective targets of anthracycline and taxane drugs, have been linked to anthracycline and taxane response in previous studies, respectively [17,18,21,42-44]. However, the expression of either of these genes has failed to become a useful clinical predictor of anthracycline or taxane response. We hypothesized that measurement of target-associated transcripts in a tumor sample might provide a more comprehensive measure of molecular target activity, and thus the tumor’s likelihood of response to therapy. Indeed, based on the datasets explored for the studies presented here, this appears to be the case. Moreover, a combination index derived from the TOP2A and β-tubulin expression indices was statistically significantly related to pathological response in a multivariate model that also included age, nodal status, tumor grade and estrogen receptor status in a group of 488 patients treated with anthracycline and taxane based chemotherapy.

From a clinical standpoint, predicting response to anthracycline and taxane based chemotherapy may be useful to identify breast cancer patients who have a high likelihood of benefiting from such regimens. Conversely, patients predicted to be resistant to anthracycline- and taxane-based chemotherapy may benefit from enrollment in clinical trials investigating the efficacy of novel treatments [45]. Many issues remain to be addressed to confirm the clinical utility of the TOP2A and β-tubulin indices. In this study our conclusions are based on the analysis of retrospective data, which limits its clinical value. Moreover, we did not establish or optimize a threshold that would serve to separate patients predicted likely to respond to therapy from those likely to be resistant. Additionally, we did not test the capacity of the TOP2A index to predict response to neoadjuvant chemotherapy that consisted of only of an anthracycline, suggesting that the TOP2A index may be predictive of general chemotherapy response. Athough we did observe that the TOP2A index was not predictive of patient response ot docetaxel. Based on our multivariate analysis, our data suggests the TOP2A and β-tubulin indices remain predictive even after adjusting for clinical parameters such as tumor grade and estrogen receptor status, indicating that these indices likely have clinical value. Strictly speaking however, a true estimate of the usefulness of these indices would require a prospective clinical trial comparing randomly selected with index selected chemotherapy regimens.

An advantage of the approach taken here is our use of publicly available data, as well as the efficient use of patient samples for validation purposes. For example, the traditional approach for gene signature identification [2,6,7,9,46], commonly called the top-down approach, multiple datasets are required that comprise both tumor gene expression profiles as well as knowledge of the clinical variables under investigation, for the purposes of signature identification and subsequent independent validation. Other approaches, such as large-scale functional based RNA interference screens, have also yielded predictive signatures, although these experiments are relatively labour intensive and expensive [47]. Here, we calculated target indices using datasets for which response to chemotherapy is not known. In this fashion, we maintained the independence of datasets for which response to neoadjuvant chemotherapy was measured as a clinical variable, thus maintaining the availability of multiple independent datasets for validation.

The identification of gene signatures that predict response to chemotherapy also have potential to offer new insights into the biology of breast tumors, particularly the transcriptional programs that govern therapy response. In this regard, it may be possible to identify molecular signaling pathways that either augment chemotherapy resistance or enhance sensitivity. Indeed, the latter strategy provides a rational approach to identifying new drug regimens, where a signaling pathway inhibitor/activator is included with the original chemotherapy regimen. In this fashion, tumors predicted to be therapy resistant might be rendered sensitive to the original therapy and treatment efficacy could be increased.

Another important implication of this study is that it highlights the identification of target based expression indices as a means to predict response to therapeutics. For example, it might be possible to generate a target based expression index for additional molecular targets, such as the HER2/Neu receptor tyrosine kinase, which is the molecular target of the humanized monoclonal antibody trastuzumab [1] as well as the small molecule Her2/Neu kinase inhibitor, lapatinib [48,49]. Using such an approach, therapeutic response to the latter agents might then be predicted using transcriptional target based signatures. Indeed, this approach could be tested for multiple new experimental molecularly targeted therapies.

## Conclusion

Importantly, these findings suggest the practicality of developing and testing target based indices that predict response to therapeutics. Moreover, our data highlights the possibility of using gene signatures to guide the use of chemotherapy during treatment of breast cancer patients.

## Methods

### Patients and Samples

All data was publicly available and downloaded from the gene expression omnibus (http://www.ncbi.nlm.nih.gov/geo/). Multiple discovery cohorts (GSE2034, GSE7390, GSE6532) were independently evaluated to determine target indices for TOP2A, β-tubulin, and E2F1. Together these cohorts comprised 811 patient tumor gene expression profiles derived from the Affymetrix U133A microarray platform (Table 2). Multiple validation cohorts were independently studied to test whether target indices were related to pathological response to neoadjuvant chemotherapy (GSE21094 (T/FAC), GSE22513 (T), GSE25055 & GSE25065 (AT). These cohorts comprised 800 patient tumor gene expression profiles from the Affymetrix U133A and Affymterix U133 Plus 2.0 microarray platforms (Table 2). The clinical characteristics of the validation cohorts are summarized in Table 3.

**Table 2:** Summary of samples used to identify and validate target indices

**Characteristic**	**Discovery cohorts**			**Validation cohorts**			
	**GSE2034**	**GSE7390**	**GSE6532**	**GSE21094**	**GSE25055**	**GSE25065**	**GSE22513**
Samples	286	198	327	278	310	198	14
Regimen	N/A	N/A	N/A	TFAC	AT	AT	T
							U133 Plus
Array type	U133A	U133A	U133A	U133A	U133A	U133A	2.0
	**Total arrays: 811**			**Total arrays: 800**			

Abbreviations: TFAC: paclitaxel/docetaxel, 5-fluorouracil, doxorubicin, cyclophosphamide; AT: doxorubicin, paclitaxel/docetaxel; T: docetaxel.

**Table 3:** Characteristics of the validation cohorts

**Characteristic**	**GSE21094**		**GSE25055**		**GSE25065**		**GSE22513**	
	**#**	**%**	**#**	**%**	**#**	**%**	**#**	**%**
**# patients**	**278**		**310**		**198**		**14**	
**Age, years**							NA	
<=50	133	48	168	54	109	55		
>50	144	52	142	46	89	45		
NA	1	0						
Mean	52		50		49			
STD	11		10		11			
**Nodal status**	NA						NA	
Positive			223	72	128	65		
Negative			87	28	71	35		
NA								
**Grade**	NA						NA	
1			19	6	13	7		
2			117	38	63	32		
3			151	49	108	55		
NA			23	7	14	7		
**Response**								
pCR	56	20	58	19	42	21	4	29
RD	222	80	248	80	140	71	10	71
NA			4	1	16	8		
**ER status**							NA	
Positive	164	49	131	42	123	62		
Negative	114	41	174	56	74	37		
NA			5	2	1	1		

Abbreviations: STD: Standard deviation; pCR: complete pathological response; RD: Residual disease. ER: Estrogen receptor; NA: Not available.

The raw intensity files (.CEL) comprising each dataset were download and normalised using the Robust Multichip Algorithm (RMA)to generate probe set intensities [50].

### Identification of Target Related Genes

Target index genes were identified by their co-expression with either TOP2A (TOP2A, 201292_at), β-tubulin (TUBB, 212320_at), or E2F1 (204947_at) based on a Pearson distance function [51]. We filtered these results such that only probe sets appearing in the most and least 1% of co- expressed probe sets within each identification cohort were included in the target index. The final TOP2A index comprised 86 probe sets with positive and 38 probe sets with negative correlation to TOP2A transcript levels (Additional file 1: Table S1). The β-tubulin index comprised 28 probe sets with positive and 14 probe sets with negative correlation to β-tubulin transcript levels (Additional file 1: Table S2).

To evaluate the target index, the expression values for each probe set were transformed such that the mean and standard deviation were set to 0 and 1 in each dataset, respectively. A target index was calculated for each patient as follows:

(1) $$\frac{\sum_{i\in P}x_{i}}{n_{P}}-\frac{\sum_{i\in N}x_{j}}{n_{N}}$$

Where x is the transformed expression, n is the number of probe sets, P is the set of probes with reported positive correlation to the target probe set, and N is the set of probes with reported negative correlation to the target probe set [46,52].

### Statistical Analysis

pCR or RD following treatment with neoadjuvant chemotherapy was used as the clinical endpoints for this study. The predictive capacities of the target indices were evaluated using receiver- operator characteristic curve (ROC) analysis and both univariate and multivariate logistic regression. T- tests were used to compare indices between responders and non-responders. Welch’s correction was used when the variance of the index was unequal in these two patient groups. All tests were two-sided and a p-value of 0.05 or less was considered statistically significant. ANOVA and Tukeys multitple comparison test was used to test for differences between multiple groups (n > 2), and p-values of 0.05 `or less were considered significant.

## Competing interests

The author’s declare no competing interests.

## Author’s contributions

Study design and conception of project, RMH and JAH. Completion of research, RMH. Statistical analysis, RMH and GP. Writing of manuscript RMH, GP and JAH. All authors read and approved the final manuscript.

## Pre-publication history

The pre-publication history for this paper can be accessed here:

http://www.biomedcentral.com/1755-8794/5/16/prepub

## Supplementary Material

Additional file 1 **Table S1 and Table S2:** Elements comprising the *TOP2A* and *β-tubulin indices.* Probe sets comprising the *TOP2A* and *β-tubulin* indices. Click here for file

Additional file 2 **Figure S1:** The E2F1 index is predictive of chemotherapy response in multiple datasets **A)** GSE21094 (n = 278, TFAC). **B)** GSE25055 (n = 310, AT). **C)** GSE25065(n = 198,AT). Click here for file
