# Supplementary figures and images for: A target based approach identifies genomic predictors of breast cancer patient response to chemotherapy
Source: BMC Med Genomics. 2012 May 11;5:16. doi: 10.1186/1755-8794-5-16 (PMC3441237; doi:10.1186/1755-8794-5-16)

## Slide 1
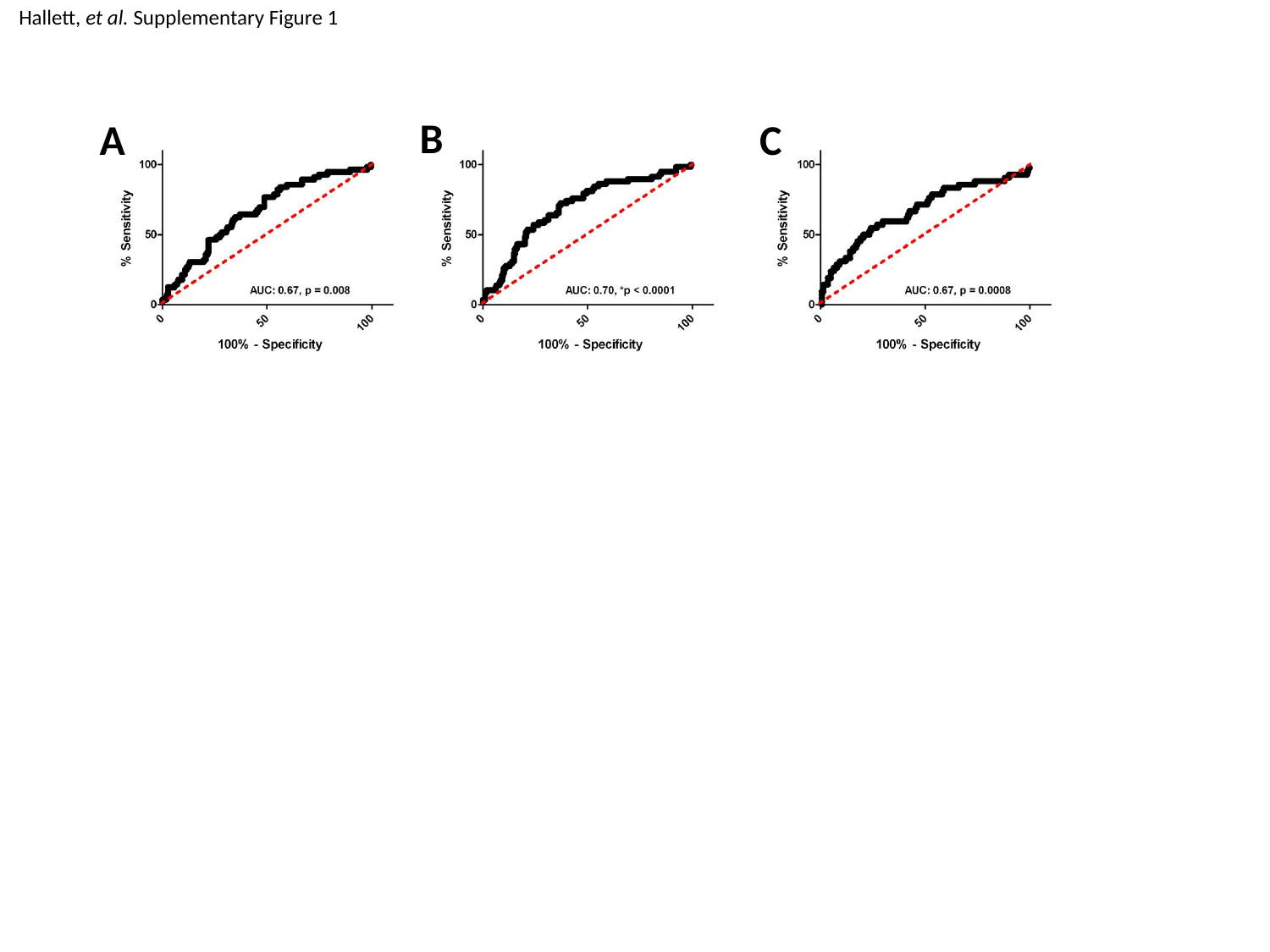

Hallett, et al. Supplementary Figure 1
B
A
C

Supplement: Additional file 2 — Figure S1: The E2F1 index is predictive of chemotherapy response in multiple datasets A) GSE21094 (n = 278, TFAC). B) GSE25055 (n = 310, AT). C) GSE25065(n = 198,AT). [file 1755-8794-5-16-S2.ppt]
